# The risk factors and predictive nomogram of human albumin infusion during the perioperative period of posterior lumbar interbody fusion: a study based on 2015–2020 data from a local hospital

**DOI:** 10.1186/s13018-021-02808-5

**Published:** 2021-10-30

**Authors:** Bo Liu, Junpeng Pan, Hui Zong, Zhijie Wang

**Affiliations:** 1 Department of Spinal Surgery, The Affiliated Hospital of Qingdao University, Qingdao, 266071 China; 2 Department of Neurology, The People’s Hospital of Qingyun, DeZhou, 253700 China

**Keywords:** Posterior lumbar interbody fusion (PLIF), Perioperative, Hypoalbuminemia, Infusion of human albumin, Multivariate logistic regression analysis, Nomogram

## Abstract

**Background:**

Perioperative hypoalbuminemia of the posterior lumbar interbody fusion (PLIF) can increase the risk of infection of the incision site, and it is challenging to accurately predict perioperative hypoproteinemia. The objective of this study was to create a clinical predictive nomogram and validate its accuracy by finding the independent risk factors for perioperative hypoalbuminemia of PLIF.

**Methods:**

The patients who underwent PLIF at the Affiliated Hospital of Qingdao University between January 2015 and December 2020 were selected in this study. Besides, variables such as age, gender, BMI, current and past medical history, indications for surgery, surgery-related information, and results of preoperative blood routine tests were also collected from each patient. These patients were divided into injection group and non-injection group according to whether they were injected with human albumin. And they were also divided into training group and validation group, with the ratio of 4:1. Univariate and multivariate logistic regression analyses were performed in the training group to find the independent risk factors. The nomogram was developed based on these independent predictors. In addition, the area under the curve (AUC), the calibration curve and the decision curve analysis (DCA) were drawn in the training and validation groups to evaluate the prediction, calibration and clinical validity of the model. Finally, the nomograms in the training and validation groups and the receiver operating characteristic (ROC) curves of each independent risk factor were drawn to analyze the performance of this model.

**Results:**

A total of 2482 patients who met our criteria were recruited in this study and 256 (10.31%) patients were injected with human albumin perioperatively. There were 1985 people in the training group and 497 in the validation group. Multivariate logistic regression analysis revealed 5 independent risk factors, including old age, accompanying T2DM, level of preoperative albumin, amount of intraoperative blood loss and fusion stage. We drew nomograms. The AUC of the nomograms in the training group and the validation group were 0.807, 95% CI 0.774–0.840 and 0.859, 95% CI 0.797–0.920, respectively. The calibration curve shows consistency between the prediction and observation results. DCA showed a high net benefit from using nomograms to predict the risk of perioperative injection of human albumin. The AUCs of nomograms in the training and the validation groups were significantly higher than those of five independent risk factors mentioned above (*P* < 0.001), suggesting that the model is strongly predictive.

**Conclusion:**

Preoperative low protein, operative stage ≥ 3, a relatively large amount of intraoperative blood loss, old age and history of diabetes were independent predictors of albumin infusion after PLIF. A predictive model for the risk of albumin injection during the perioperative period of PLIF was created using the above 5 predictors, and then validated. The model can be used to assess the risk of albumin injection in patients during the perioperative period of PLIF. The model is highly predictive, so it can be clinically applied to reduce the incidence of perioperative hypoalbuminemia.

## Introduction

As the population ages, the incidence of lumbar degenerative disc diseases such as herniated disk, lumbar spinal stenosis and degenerative spondylolisthesis increased year by year [1]. Low back pain and sciatica are common disabling diseases, which have a significant impact on patients' social, work and economic life [2]. Posterior lumbar interbody fusion (PLIF) is now the most common surgical procedure for these diseases [3, 4]. Several types of cages, such as metal cages and polyetheretherketone (PEEK) cages, were used in the TLIF procedure [5]. Long operative time, large incision size, and severe bleeding can not be ignored, despite its advantages of good curative effect and low recurrence rate. Complications such as cerebrospinal fluid (CSF) leak, wound infection, anemia, and hypoalbuminemia are commonly seen in the perioperative period [6].

Hypoalbuminemia is a common complication of PLIF. It can increase the risk of nonunion, infection and lower limb edema after PLIF. Hypoalbuminemia is associated with inflammation. It can increase the muscle mass and function, but this loss is accelerated by comorbidity and associated with decreasing serum albumin levels [7]. So this raises increasing concerns of surgeons in the perioperative period [8]. Albumin is a protein synthesized by the liver and plays a role in maintaining oncotic pressure and improving immunity. Human serum albumin has been widely used in various areas of the clinic for nearly 70 years. Although there is no evidence to support the use of albumin over crystalloids in acute volume resuscitation, many clinicians continue to use albumin because it has other important physiological roles in addition to tumor function [9]. Albumin ≤ 28 g/L in liver function was seen as an indicator of the need for perioperative injection of human albumin, which can increase the level of plasma albumin in a short time. So this can improve immunity and help recovery [10].

Nomogram is widely used in the diagnosis and prognosis of diseases. It can integrate multiple risk factors to make a comprehensive assessment of the risk of diseases, and visualize the results to make them easy to understand [11]. It has been suggested that blood transfusion risk models during posterior lumbar interbody fusion (PLIF) surgery are beginning to be established, which provide a reference for clinical prevention and reducing the occurrence of perioperative blood transfusion [12]. At present, there are few studies on the risk factors of infusion of human albumin during the perioperative period of PLIF. And no researchers have created a relevant predictive model. So this study aims to build a clinically predictive model by finding the incidence and risk factors of albumin infusion during the perioperative period of PLIF and validate the model.

## Methods

### Collection of patients’ information

The information of patients who underwent PLIF at the Department of Spine of The Affiliated Hospital of Qingdao University between January 2015 and December 2020 was collected. This study was also approved by the Ethics Committee of the Affiliated Hospital of Qingdao University. Since our study is a retrospective analysis, the Ethics Committee particularly approved that informed consent by each participant was not required. There are two inclusion criteria, including (1) patients being diagnosed with lumbar degenerative disc diseases, such as herniated disk, spondylolisthesis and lumbar spinal stenosis; and (2) patients having undergone PLIF. There are also two exclusion criteria, including (1) patients with severe complications such as thrombosis, paralysis, and death during or within 3 days after surgery and (2) patients with incomplete clinical data.

The conditions that patients need to meet for transfusion of human albumin: patients with low albumin symptoms whose perioperative reexamination of liver function suggests albumin 28 g/L or albumin ≤ 30 g/L. Currently, there is no consensus among researchers on the condition that patients need to meet for injection of human albumin during the perioperative period. But individualized management is necessary for each patient during this period. And we try to predict the time when albumin should be injected as precisely as we could.

The data collected included demographic characteristics, past medical history, accompanying diseases, indications for surgery, results of preoperative liver function tests, operative time, amount of bleeding, and fusion stage of each patient, as shown in Table 1. All data were independently collected by two spine surgeons from our hospital’s medical records system, and any disputed data were modified with the consent of the two surgeons. PLIF was the operation that was performed. Surgeons that operated on the patients were all senior doctors. Each patient was clinically managed on a unified basis.

**Table 1:** Demographic characteristics of infused and non infused human albumin

	Infused human albumin		*P*
	No (*N* = 2226)	Yes (*N* = 256)	
Age (%)			< 0.001
< 55	344 (15.5)	36 (14.1)	
55–75	1606 (72.1)	143 (55.9)	
> 75	276 (12.4)	77 (30.1)	
Sex (%)			0.572
Male	1084 (48.7)	130 (50.8)	
Female	1142 (51.3)	126 (49.2)	
BMI (mean (SD))	25.69 (3.58)	25.05 (4.20)	0.008
Previous history (%)			
Surgery	484 (21.7)	48 (18.8)	0.305
Blood transfusion	58 (2.6)	6 (2.3)	0.966
Allergies	41 (1.8)	5 (2.0)	0.905
Smoking	376 (16.9)	38 (14.8)	0.457
Alcohol consumption	326 (14.6)	31 (12.1)	0.317
Comorbidities (%)			
Hypertension	754 (33.9)	98 (38.3)	0.181
T2DM	438 (19.7)	77 (30.1)	< 0.001
Coronary heart disease	313 (14.1)	37 (14.5)	0.940
Cerebral thrombosis	18 (0.8)	5 (2.0)	0.143
Respiratory diseases	123 (5.5)	15 (5.9)	0.939
Digestive system diseases	212 (9.5)	38 (14.8)	0.010
Other	294 (13.2)	33 (12.9)	0.965
Laboratory tests (median [IQR])			
TP	75.11 [65.80, 100.00]	81.44 [65.86, 100.00]	0.804
GLO	24.90 [22.20, 27.37]	25.26 [22.58, 27.45]	0.364
ALB	36.51 [31.40, 46.70]	28.56 [21.23, 36.85]	< 0.001
ALB/GLO	1.44 [1.14, 1.79]	1.04 [0.73, 1.41]	< 0.001
TBIL	13.10 [9.91, 17.30]	13.27 [9.80, 17.11]	0.544
DBIL	3.63 [2.66, 5.00]	3.70 [2.50, 5.00]	0.800
IBIL	9.48 [7.05, 12.41]	9.30 [7.04, 12.00]	0.497
ALT	18.70 [14.00, 28.00]	19.00 [15.07, 28.00]	0.134
AST	17.30 [14.40, 22.00]	18.00 [15.00, 22.12]	0.249
ALT/AST	1.10 [0.86, 1.42]	1.10 [0.83, 1.40]	0.466
PA	219.00 [176.00, 262.00]	204.65 [165.62, 251.40]	0.005
Operation (median [IQR])			
Bleeding volume	400.00 [400.00, 550.00]	400.00 [400.00, 600.00]	0.001
Surgery time	140.00 [100.00, 200.00]	157.50 [115.00, 210.00]	0.005
Indications for surgery (%)			
LDH	1294 (58.1)	145 (56.6)	0.575
LSS	803 (36.1)	92 (35.9)	
Lumbar spondylolisthesis	129 (5.8)	19 (7.4)	
Fusion segment (%)			
1	1024 (46.0)	97 (37.9)	< 0.001
2	881 (39.6)	92 (35.9)	
≥ 3	321 (14.4)	67 (26.2)	

### Statistical analysis

All statistical analyses were done through SPSS (version 26, IBM, USA) and R software (version 4.0.3, R Foundation for statistical Computing, Austria). The normality of continuous variables was determined by the Shapiro–Wilk test in SPSS. The value of normal distribution is expressed as mean ± SD, and the value of non-normal distribution as medians (interquartile ranges). Categorical variables are expressed as counts and percentages. Continuous variables were tested by Student’s t test (normal) or Mann–Whitney U test (non-normal), and categorical variables by chi-square test. In this study, *P* < 0.05 (bilateral) is statistically significant.

All patient data were divided into training group and validation group, with the ratio of 3:1 using R software (version 4.0.3, R Foundation for statistical Computing, Austria). The data of the training group were used for building the model and those of validation group for validating the prediction of the model. First, we performed a univariate logistic regression analysis in the training group to find the factors associated with the infusion of albumin during the perioperative period of PLIF. Then, multivariate logistic regression analysis was carried out to determine the independent risk factors of perioperative infusion. Finally, a nomogram was created with the results of multivariate logistic regression analysis. ROC curves, calibration curves and the DCAs were drawn for both the training and validation groups.

The area under the curve (AUC) of ROC represents the degree of the predictive performance of the model. The AUC ranges from 0.5 to 1.0. The closer the value of AUC is to 1.0, the better the model is at predicting. The calibration curve was a comparison image between the predicted risk and the real risk for the patients. The more consistent the predicted risks are with the calibration curve, the better the model fits the data. DCA was used to evaluate the net benefit and effectiveness of the model, and calculate the net benefit. Finally, the nomograms for the training and validation groups and ROC curve of each independent risk factor were drawn to analyze the prediction of nomograms and each independent predictor.

## Results

### The demographic characteristics of the patients

A total of 2, 482 patients were enrolled and 256 patients (10.31%) were perioperatively injected with albumin. A totao of 276 patients aged over 75 were not transfused with albumin. And there were 1084 (48.7%) males, 1142 (51.3%) females and 438 (19.7%) diabetics. Besides, the preoperative albumin level of 36.51 [31.40, 46.70], the intraoperative blood loss of 400.00 ML [400.00, 550.00] and 321 (14.4%) patients with fusion stage ≥ 3 were found. Seventy-seven patients aged over 75 were transfused with human albumin. And there were 130 (50.8%) males, 126 (49.2%) females and 77 (30.1%) diabetics. Besides, the preoperative albumin level of 28.56 [21.23, 36.85], the intraoperative blood loss of 400.00 ML [400.00, 600.00] and 67 (26.2%) patients with fusion stage ≥ 3 were found (Table 1).

### Analysis of relevant and independent risk factors in training set

A total of 1, 985 patients were enrolled in the training group and 205 patients were injected with human albumin (Table 2). The univariate regression analysis showed 10 variables with P-value less than 0.05, including old age, BMI, accompanying T2DM and gastrointestinal disease, preoperative albumin, the ratio of albumin to globulin, prealbumin, blood loss, operative time, and stage of fusion (Table 3). Multivariate logistic regression analysis revealed 5 independent risk factors, including old age, accompanying T2DM, preoperative albumin, intraoperative blood loss and fusion stage (Table 3).

**Table 2:** Demographic characteristics of human albumin infusion and non infusion in training population and validation population

	Training cohort		Test cohort	
	No (*N* = 1780)	Yes (*N* = 205)	No (*N* = 446)	Yes (*N* = 51)
Age (%)				
< 55	283 (15.5)	29 (13.9)	61 (15.3)	7 (14.9)
55–75	1330 (72.8)	126 (60.3)	276 (69.0)	17 (36.2)
> 75	113 (11.7)	54 (25.8)	63 (15.8)	23 (48.9)
Sex (%)				
Male	855 (48.1)	103 (49.3)	229 (50.9)	27 (57.4)
Female	921 (51.9)	106 (50.7)	221 (49.1)	20 (42.6)
BMI (mean (SD))	25.67 (3.59)	25.14 (4.22)	25.76 (3.51)	24.62 (4.12)
Previous history (%)				
Surgery	403 (22.7)	39 (18.7)	81 (18.0)	9 (19.1)
Blood transfusion	43 (2.4)	5 (2.4)	15 (3.3)	1 (2.1)
Allergies	25 (1.4)	4 (1.9)	16 (3.6)	1 (2.1)
Smoking	294 (16.6)	35 (16.7)	82 (18.2)	3 (6.4)
Alcohol cconsumption	245 (13.8)	27 (12.9)	81 (18.0)	4 (8.5)
Comorbidities disease (%)				
Hypertension	595 (33.5)	76 (36.4)	159 (35.3)	22 (46.8)
T2DM	347 (19.5)	63 (30.1)	91 (20.2)	14 (29.8)
Coronary heart disease	254 (14.3)	31 (14.8)	59 (13.1)	6 (12.8)
Cerebral thrombosis	14 (0.8)	2 (1.0)	4 (0.9)	3 (6.4)
Pneumonia	99 (5.6)	12 (5.7)	24 (5.3)	3 (6.4)
Chronic gastritis	161 (9.1)	28 (13.4)	51 (11.3)	10 (21.3)
Other	237 (13.3)	27 (12.9)	57 (12.7)	6 (12.8)
Laboratory tests (median [IQR])				
TP	75.00 [65.79, 100.00]	99.80 [65.31, 100.00]	76.14 [66.11, 100.00]	74.70 [67.61, 100.00]
GLO	24.80 [22.19, 27.30]	25.26 [22.40, 27.20]	25.26 [22.50, 27.73]	25.26 [23.09, 27.83]
ALB	36.79 [31.40, 46.90]	29.10 [21.00, 38.00]	36.16 [31.42, 46.20]	26.51 [21.80, 33.85]
ALB GLO	1.44 [1.15, 1.80]	1.04 [0.75, 1.44]	1.40 [1.10, 1.77]	0.94 [0.71, 1.25]
TBIL	13.10 [10.00, 17.29]	13.29 [9.91, 17.13]	13.14 [9.79, 17.43]	13.12 [9.47, 16.50]
DBIL	3.67 [2.64, 5.00]	3.74 [2.65, 5.00]	3.60 [2.75, 4.91]	3.37 [2.37, 5.07]
IBIL	9.45 [7.07, 12.40]	9.30 [7.20, 12.00]	9.60 [7.00, 12.44]	9.67 [6.50, 11.96]
ALT	18.85 [14.00, 28.00]	19.30 [15.20, 29.80]	18.70 [13.83, 28.00]	17.90 [15.00, 24.00]
AST	17.25 [14.38, 21.33]	18.00 [15.00, 22.60]	17.45 [14.50, 23.00]	18.00 [14.70, 21.50]
ALT/AST	1.10 [0.86, 1.42]	1.13 [0.84, 1.40]	1.08 [0.83, 1.40]	0.92 [0.78, 1.30]
PA	220.30 [176.00, 261.50]	205.00 [165.80, 251.80]	210.70 [177.18, 262.80]	192.40 [165.95, 222.65]
Surgery (median [IQR])				
Bleeding Volume	400.00 [400.00, 550.00]	400.00 [400.00, 600.00]	400.00 [400.00, 550.00]	500.00 [400.00, 600.00]
Surgery time	140.00 [100.00, 200.00]	155.00 [115.00, 210.00]	140.00 [100.00, 200.00]	160.00 [125.00, 207.50]
Surgical indications (%)				
Lumbar spondylolisthesis	109 (6.1)	16 (7.7)	20 (4.4)	3 (6.4)
LDH	1034 (58.2)	122 (58.4)	260 (57.8)	23 (48.9)
LSS	633 (35.6)	71 (34.0)	170 (37.8)	21 (44.7)
Fusion segment (%)				
1	826 (46.4)	75 (36.4)	198 (44.3)	22 (44.0)
2	693 (39.0)	78 (37.9)	188 (42.1)	14 (28.0)
≥ 3	260 (14.6)	53 (25.7)	61 (13.6)	14 (28.0)

**Table 3:** Univariate and multivariate analysis of human albumin infusion in PLIF Perioperative period

	Univariable regression	*P* value	Multivariable regression	*P* value
Age				
< 55	Ref		Ref	
55–75	0.962 (0.633, 1.464)	0.857	0.929 (0.584, 1.476)	0.359
> 75	6.041 (3.476, 0.499)	** < 0.001**	5.610 (2.950, 10.667)	** < 0.001**
Sex				
Male	0.955 (0.717, 1.273)	0.755		
Female	Ref			
BMI	0.96 (0.923, 1.000)	**0.049**	0.977 (0.935, 1.021)	0.323
Previous history				
Surgery	0.782 (0.542, 1.126)	0.186		
Blood transfusion	0.988 (0.387, 2.522)	0.980		
Allergies	1.367 (0.471, 3.966)	0.566		
Smoking	1.014 (0.691, 1.489)	0.944		
Alcohol	0.927 (0.605, 1.420)	0.727		
Comorbidities disease				
Hypertension	1.134 (0.841, 1.529)	0.408		
T2DM	1.777 (1.293, 2.442)	** < 0.001**	2.143 (1.493, 3.075)	** < 0.001**
Coronary heart	1.044 (0.697, 1.563)	0.836		
Cerebral thrombosis	1.216 (0.274, 5.388)	0.797		
Respiratory	1.032 (0.557, 1.912)	0.921		
Digestive system	1.552 (1.010, 2.385)	**0.045**	1.607 (0.974, 2.651)	0.050
Other	0.963 (0.629, 1.476)	0.864		
Laboratory tests				
TP	1.003 (0.995, 1.011)	0.491		
GLO	1.014 (0.977, 1.052)	0.458		
ALB	0.867 (0.847, 0.886)	** < 0.001**	0.873 (0.844, 0.903)	** < 0.001**
ALB/GLO	0.108 (0.072, 0.163)	** < 0.001**	0.883 (0.477, 1.634)	0.337
TBIL	0.992 (0.969, 1.016)	0.511		
DBIL	0.996 (0.933, 1.064)	0.915		
IBIL	0.986 (0.954, 1.019)	0.397		
ALT	1.004 (0.998, 1.009)	0.203		
AST	1.006 (0.995, 1.017)	0.276		
ALT/AST	0.928 (0.707, 1.217)	0.587		
PA	0.997 (0.995, 1.000)	**0.023**	0.998 (0.995, 1.000)	0.202
Surgery				
Bleeding volume	1.001 (1.001, 1.002)	** < 0.001**	1.001 (1.000, 1.002)	** < 0.001**
Time	1.002 (1.000, 1.004)	**0.030**	1.002 (1.000, 1.004)	0.090
Indications				
LDH	0.804 (0.460, 1.403)	0.442		
LSS	0.764 (0.428, 1.364)	0.363		
Spondylolisthesis	Ref			
Fusion segment				
1	Ref		Ref	
2	1.240 (0.889, 1.729)	0.206	1.127 (0.779, 1.630)	0.526
≥ 3	2.245 (1.538, 3.277)	** < 0.001**	1.734 (1.117, 2.691)	**0.014**

Bold indicates that the* p* value of these variables is less than 0.05, which is statistically significant

### Development and validation of nomogram to predict the risk of perioperative infusion of human albumin

**Fig. 1:**
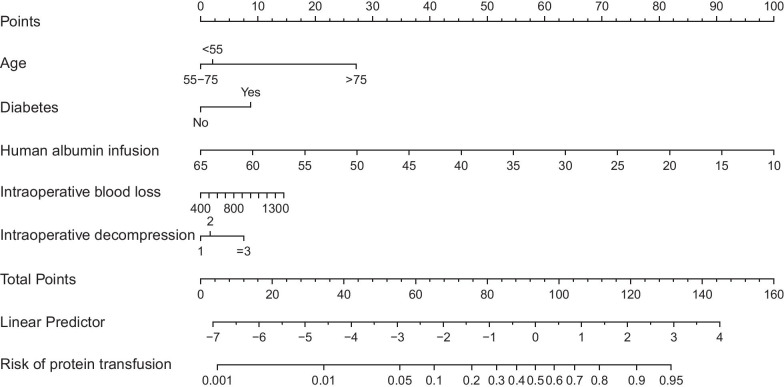
A nomogram for predicting perioperative transfusion of human albumin

**Fig. 2:**
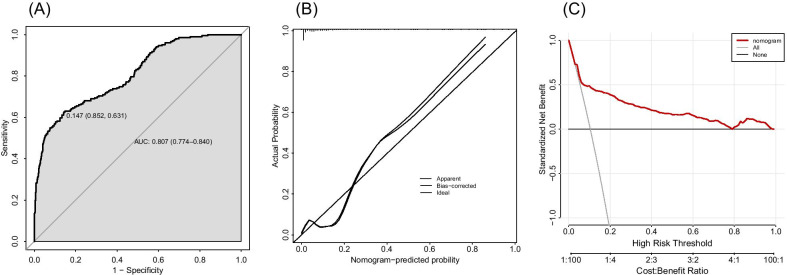
**a** ROC curve in training set to evaluate prediction accuracy; **b** calibration curve in training set; **c** decision curve analysis in training set

Five independent predictors including old age, accompanying T2DM, preoperative albumin, intraoperative blood loss and fusion stage were used to draw the nomogram (Fig. 1). In the training set, the AUC of the nomogram was 0.807, 95% CI 0.774–0.840. High-accuracy in predicting the risk of albumin infusion during the perioperative period of PLIF can be seen (Fig. 2a). In addition, the calibration curve shows good consistency between the predicted and observed results (Fig. 2b). DCA showed a high net benefit from using nomograms to predict the risk of perioperative injection of human albumin (Fig. 2c).

**Fig. 3:**
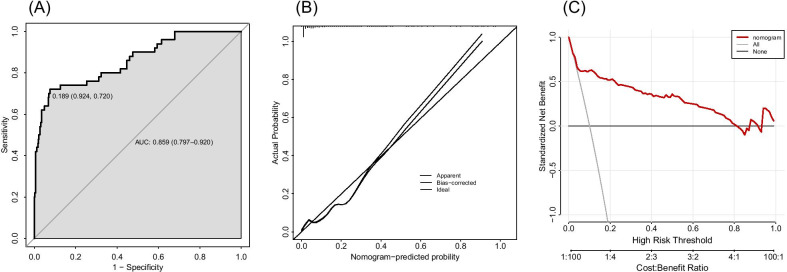
**a** ROC curve in test set to evaluate prediction accuracy; **b** calibration curve in test set. **c** Decision curve analysis in test set

In the study, 497 patients were enrolled, 51 of whom were perioperatively injected with human albumin (Table 2). The AUC of nomograms for predicting the probability of transfusion was 0.859, 95% CI 0.797–0.920 (Fig. 3a). The calibration curve showed high-consistency between predicted and observed blood transfusion probability (Fig. 3b). In addition, DCA proved that the model had a higher net benefit (Fig. 3c).

### Evaluation of the performance of the predictive model

**Fig. 4:**
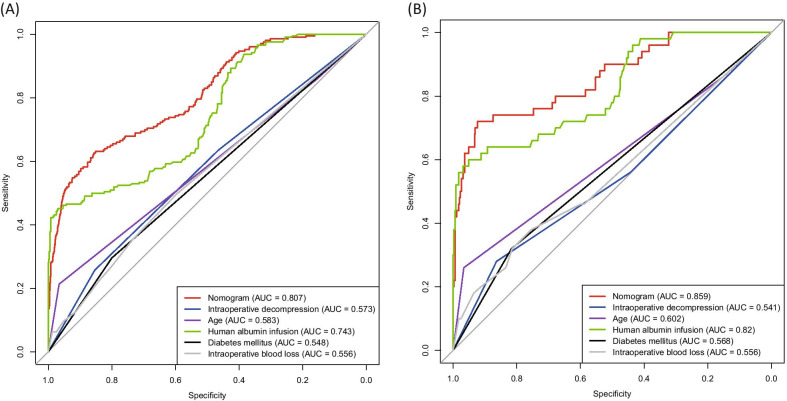
Comparison of the predictive power of different indicators and nomogram plots for transfusion risk in training (**a**) and test datasets (**b**)

Six ROC curves of nomogram model, old age, accompanying T2DM, preoperative albumin, intraoperative blood loss, and fusion stage were drawn using the training set (Fig. 4a). The results show that AUCs of nomograms were significantly higher than those of the five independent risk factors above (*P* < 0.001). Similarly, the ROC curve in the test set was drawn. The AUC of nomogram was also significantly higher than that of each independent risk factor in the test set (*P* < 0.001) (Fig. 4b). So it can be concluded that a clinically predictive model for the risk of human albumin infusion during the perioperative period of PLIF has good performance. It is highly predictive, so it can be widely clinically applied.

## Discussion

PLIF is the primary surgical procedure for the treatment of lumbar degenerative disc diseases. During the operation, it is necessary to remove more muscle tissue, implant pedicle screw, remove the intervertebral disc and deal with the surface of bone graft after fusion to scrape out cartilage endplate. Long operative time, large incision size and severe blood loss are the defects of this surgery [13]. In addition, severe inflammatory response and a relatively large amount of drainage from incision can be usually found in perioperative patients. This makes these patients more likely to get hypoalbuminemia which is treated primarily by the infusion of albumin [14].

In this study, the incidence of hypoalbuminemia in the perioperative period was about as high as 10.31%, and albumin ≤ 28G/L was taken as the standard for grouping. Data of 573 cases who were surgically treated for fractures of the lower extremities (including the pelvis and acetabulum) were collected from ACS-NSQIP database by Wilson and other researchers. And up to 29.6% of them have hypoalbuminemia. This is mainly because the subjects in the study group were older than 65.

The results of multivariate logistic regression analysis show that the independent risk factors of albumin infusion during the perioperative period of PLIF were stage ≥ 3, severe intraoperative blood loss, preoperative low level of protein, old age, and history of diabetes. Besides, the nomograms are drawn. Preoperative hypoalbuminemia is one of the independent risk factors. Albumin, a protein produced by the liver, has the function of transporting substances and maintaining plasma oncotic pressure. Hypoalbuminemia causes a decrease in capillary filtration pressure, leading to tissue edema due to extravasation of tissue fluid and then the delayed wound healing [7, 15, 16]. Besides, the albumin metabolism cycle is longer and half-life lasts 15–19 days. Patients with preoperative low level of albumin showed malnutrition or diminished strength, making them less tolerate surgery [17–19]. More than 3 decompressed segments and severe intraoperative blood loss were independent risk factors for perioperative hypoalbuminemia. More decompressed segments entail more time for the surgery and more muscle tissue discectomy removed, resulting in more intraoperative blood loss and postoperative drainage. Besides, patients with larger surgical incision will suffer severe inflammatory response, causing more albumin to lose.

Elderly patients during the perioperative period of PLIF are likely to get hypoalbuminemia, which is usually accompanied by other systemic diseases and a decrease in organ activities, especially in the ability of the liver to produce and metabolize albumin [20, 21]. The postoperative stress response to surgery for many old people is more severe than that of young people. The symptoms are nausea and vomiting after general anesthesia [22–24]. This leads to decreased digestive and absorption functions of the gastrointestinal tract, resulting in loss of appetite and eating less. This leads to a reduction in protein intake, and then low level of albumin. Diabetes is an independent risk factor for PLIF, which may be associated with diabetes-induced vascular permeability changes and perioperative proteinuria, leading to increased albumin loss [25–27]. But some studies show that diabetes is not an independent risk factor for postoperative hypoalbuminemia. So this issue needs to be further studied.

This model can significantly and clinically improve the level of prediction and diagnosis of perioperative hypoalbuminemia, thus providing individualized treatment for each patient. Improved prediction and diagnosis, for example, can help identify high-risk patients and tell them to increase the intake of protein at admission to the hospital and make early postoperative dietary adjustments. If necessary, human albumin needs to be injected. Using this nomogram can help us accurately calculate the probability of hypoalbuminemia in each patient during the perioperative period. For those patients who have a higher risk of hypoalbuminemia in this period, surgeons can take preventive measures in advance to prevent more serious complications [28–31]. No researchers have developed and validated a risk model for hypoalbuminemia during the perioperative period of PLIF.

However, clinical predictive models can not predict the occurrence of perioperative hypoalbuminemia with 100 percent accuracy, and our nomogram has some limitations. First, this is a clinical retrospective study, where we increased the sample size while introducing more possible factors to minimize selection bias. Second, the data we collected were from a local, single-center hospital. So different regions, ethnic groups, and races could be the factors influencing the accuracy of the predictive model. Finally, we did not use all the study variables, so there may be missing on important variables. We assume that a bigger sample size and multi-center research will make the prediction of the model more reliable and persuasive.

## Conclusion

Preoperative low level of protein, operative stage ≥ 3, severe intraoperative blood loss, old age and history of diabetes were independent predictors of albumin infusion in the postoperative period of PLIF. So these five predictors can help predict the risk of infusion of albumin. And a predictive model to predict the risk of injecting albumin perioperatively was created and then validated. This infusion model can be used to assess the risk of albumin infusion for patients in the perioperative period of PLIF. The model is highly predictive, so it can be clinically applied to reduce the incidence of perioperative hypoalbuminemia.

## Data Availability

The data used and analyzed during the current study are available in anonymized form from the corresponding author on reasonable request.

## Abbreviations

PLIF: Posterior lumbar interbody fusion
CSF: Complications such as cerebrospinal fluid
ROC: Receiver operating characteristic
OR: Odds ratio
AUC: Area under the curve
CI: Confidence interval
